# Type 3 diabetes and metabolic reprogramming of brain neurons: causes and therapeutic strategies

**DOI:** 10.1186/s10020-025-01101-z

**Published:** 2025-02-18

**Authors:** Xiangyuan Meng, Hui Zhang, Zhenhu Zhao, Siyao li, Xin Zhang, Ruihan Guo, Huimin Liu, Yiling Yuan, Wanrui Li, Qi Song, Jinyu Liu

**Affiliations:** 1 Department of Toxicology, School of Public Health, Jilin University, Changchun, 130021 China; 2 Institute of Agricultural Quality Standard and Testing Technology, Jilin Academy of Agricultural Sciences, Changchun, 130021 China

**Keywords:** Alzheimer’s disease, Type 3 diabetes mellitus, Insulin resistance, Metabolic reprogramming

## Abstract

Abnormal glucose metabolism inevitably disrupts normal neuronal function, a phenomenon widely observed in Alzheimer’s disease (AD). Investigating the mechanisms of metabolic adaptation during disease progression has become a central focus of research. Considering that impaired glucose metabolism is closely related to decreased insulin signaling and insulin resistance, a new concept "type 3 diabetes mellitus (T3DM)" has been coined. T3DM specifically refers to the brain’s neurons becoming unresponsive to insulin, underscoring the strong link between diabetes and AD. Recent studies reveal that during brain insulin resistance, neurons exhibit mitochondrial dysfunction, reduced glucose metabolism, and elevated lactate levels. These findings suggest that impaired insulin signaling caused by T3DM may lead to a compensatory metabolic shift in neurons toward glycolysis. Consequently, this review aims to explore the underlying causes of T3DM and elucidate how insulin resistance drives metabolic reprogramming in neurons during AD progression. Additionally, it highlights therapeutic strategies targeting insulin sensitivity and mitochondrial function as promising avenues for the successful development of AD treatments.

## Introduction

Alzheimer's disease (AD) is one of the most pressing public health challenges in the context of global population aging. By 2030, the number of individuals with AD is expected to increase to 82 million, placing a significant burden on global healthcare systems (2024b). Currently, two FDA-approved drugs, Lecanemab and Donanemab, directly target the removal of β-amyloid (Aβ) plaques and have shown promising results in slowing the progression of cognitive decline in AD. However, they do not exempt from unwanted secondary effects (Huang et al. 2023c; Huimin et al. 2023; Sims et al. 2023; van Dyck et al. 2023). Despite receiving treatment, patients continue to experience disease progression. These findings underscore the critical importance of advancing our understanding of the pathogenesis of AD and identifying potential therapeutic targets.

Data from epidemiological and clinical studies support the notion that obesity or diabetes, which lead to insulin resistance, are significant risk factors for AD (Ahtiluoto et al. 2010; Profenno et al. 2010; Talbot et al. 2012). Given the brain’s high sensitivity to insulin, peripheral insulin resistance results in reduced insulin signaling in the central nervous system (CNS), subsequently leading to alterations in brain metabolism (Kapogiannis and Avgerinos 2020). Increasing evidence suggests that Aβ toxicity, tau hyperphosphorylation, oxidative stress, and neuroinflammation are attributed to CNS insulin resistance, thereby contributing to neurodegeneration (Takeda et al. 2012; Wei et al. 2021; Leclerc et al. 2022). Considering the shared molecular and cellular features between type 1 diabetes, type 2 diabetes mellitus (T2DM), and insulin resistance in older adults, which are associated with memory impairment and cognitive decline, researchers have coined the term “Type 3 Diabetes mellitus (T3DM)” to emphasize the critical role of insulin in brain energy supply (Steen et al. 2005).

The brain is a highly energy-demanding organ, with mitochondria providing sufficient energy and meeting its energy needs through oxidative phosphorylation (OXPHOS). Glucose is the primary energy source for neurons, facilitating the production of more than 95% of adenosine triphosphate (ATP) through glycolysis and OXPHOS (Kapogiannis and Avgerinos 2020). While ATP can be generated through glycolysis alone, glycolysis produces only two ATP molecules, which are insufficient to meet the energy demands of neurons. This process also leads to the accumulation of reactive oxygen species (ROS). Consequently, any factor that impairs the normal OXPHOS process in neurons results in energy depletion, which can trigger neuronal death and ultimately lead to the onset of neurodegenerative diseases. Studies have shown that, before the onset of cognitive impairment, alterations in glucose metabolism pathways occur in the neurons of the brains of AD patients (Wei et al. 2023). This suggests that during the progression of AD, neurons undergo a metabolic reprogramming process, shifting from OXPHOS to glycolysis. Additionally, ATP production is reduced in the brains of at least 7% of early-onset AD, 20% of late-onset AD, and 35%-50% of advanced AD patients. Notably, the reduction in energy synthesis in neurons occurs before the onset of cognitive decline and pathological features of AD. As the disease progresses, this suggests that energy synthesis dysfunction may be one of the early characteristic pathological changes in AD (Hoyer 1992). However, the complexity of brain metabolism indicates that these findings should be interpreted with caution, as metabolic shifts may become maladaptive over time, activating a series of complex compensatory responses that contribute to the progression of AD.

Given the association between reduced brain insulin levels or insulin receptor signaling and cognitive dysfunction, as well as neurodegenerative diseases, the mechanisms involving central insulin signaling, glucose utilization, and neuronal energy homeostasis—particularly the metabolic reprogramming of neuronal energy metabolism—are emerging as promising areas of research and intervention (Chang et al. 2020; Milstein and Ferris 2021; Huang et al. 2023a;Miranda 2024). In this review, we explore the potential mechanisms underlying the induction of T3DM and how insulin resistance drives neuronal metabolic reprogramming. We also summarize therapeutic strategies for addressing T3DM.

### Insulin resistance and AD

AD is the most common cause of dementia, accounting for 60–80% of all dementia cases. It is a distinct neurodegenerative disease characterized by the accumulation of amyloid plaques and tau tangles in the brain, leading to progressive cognitive decline. As a progressive neurodegenerative disorder with an insidious onset, AD is rapidly becoming a major global burden on healthcare, society, and the economy (2024b). According to the 2022 World Alzheimer’s Report, the number of individuals affected by AD is expected to exceed 100 million by 2050, placing a substantial strain on global healthcare systems (Serge Gauthier 2022).

Several widely accepted hypotheses have been proposed to explain the pathogenesis of AD, including the amyloid cascade hypothesis, the cholinergic hypothesis, the hypothesis of tau hyperphosphorylation, the neuroinflammation hypothesis, and the metal ion dysregulation hypothesis. However, the exact mechanisms underlying the disease remain unclear. Current treatments for AD, such as Donepezil, Rivastigmine, and Galantamine, are acetylcholinesterase inhibitors approved by the Food and Drug Administration (FDA) for symptom management (Rogers and Friedhoff 1996; Rösler et al. 1999; Raskind et al. 2000). In 2003, the non-competitive *N*-methyl-d-aspartate (NMDA) receptor (NMDAR) antagonist Namenda was also FDA-approved (Areosa and Sherriff 2003). Namzaric, a combination of Donepezil and Namenda, was approved in 2014 (Howard et al. 2012). However, these treatments primarily target cognitive symptoms such as memory loss and confusion. They do not alter the progression of the disease or address the underlying neurodegenerative processes. In recent years, the FDA has approved new AD treatments, including Aducanumab and Lecanemab, developed by Biogen/Esai (Budd Haeberlein et al. 2022; van Dyck et al. 2023). However, clinical trials for Aducanumab showed efficacy only in high-dose groups, while Lecanemab has notable side effects, such as amyloid-related imaging abnormalities, including brain edema and cerebral hemorrhage (Wu et al. 2023). While both Aducanumab and Lecanemab aim to clear amyloid plaques in the brain, their side effects and clinical efficacy still require extensive monitoring. Given the complexity of AD, it is critical to explore its pathogenic mechanisms and identify potential therapeutic targets.

### Brain energy supply and insulin resistance

Although the human brain constitutes just over 2% of body weight, it consumes approximately 20% of the body’s total energy demand (Goyal et al. 2014). Neurons primarily rely on oxidative metabolism, using glucose as their main energy source, and employ OXPHOS to provide sufficient energy for synaptic transmission and the maintenance of neuronal functions (Lin and Beal 2006; Koopman et al. 2013). As a result, glucose provides the vast majority of calories consumed by the adult brain. Most glucose is oxidized to produce the substantial amounts of ATP needed to maintain membrane ion gradients and other cellular processes involved in synaptic transmission (Kapogiannis and Avgerinos 2020). Maintaining glucose homeostasis requires both hormonal and neural regulation, which supports the proper functioning of the brain and peripheral tissues. Glucose is not only the primary energy source for both neural and non-neural cells, but it also serves as a signaling molecule (Li et al. 2020). For example, AMP-activated protein kinase (AMPK) responds to changes in the intracellular AMP/ATP and/or ADP/ATP ratios, thereby regulating mTORC1 activity. This signaling pathway coordinates cell growth, proliferation, metabolism, and survival with the cell’s nutritional environment (Zhou and Liu 2022). Thus, glucose regulation mechanisms are essential to ensuring an adequate supply of glucose to meet the metabolic demands of both the central nervous system and peripheral tissues. Insulin is a key hormone that regulates blood glucose absorption and promotes anabolic metabolism, facilitating the synthesis of glycogen, fats, and proteins (Petersen and Shulman 2018). Under normal conditions, insulin-sensitive organs or tissues (such as the brain, skeletal muscles, liver, and adipose tissue) require endogenous or exogenous insulin at lower concentrations to elicit a physiological response. However, in the presence of insulin resistance, these tissues require higher concentrations of insulin to respond to its effects. This condition results in a decreased efficiency of insulin in glucose uptake and utilization, clinically defined as insulin resistance (Petersen and Shulman 2018). Insulin resistance is considered a driving factor in the pathogenesis of many modern diseases, including metabolic syndrome, non-alcoholic fatty liver disease (NAFLD), atherosclerosis, T2DM, and neurodegenerative diseases (Zhao et al. 2023).

#### Insulin and the insulin signaling pathway

**Fig. 1:**
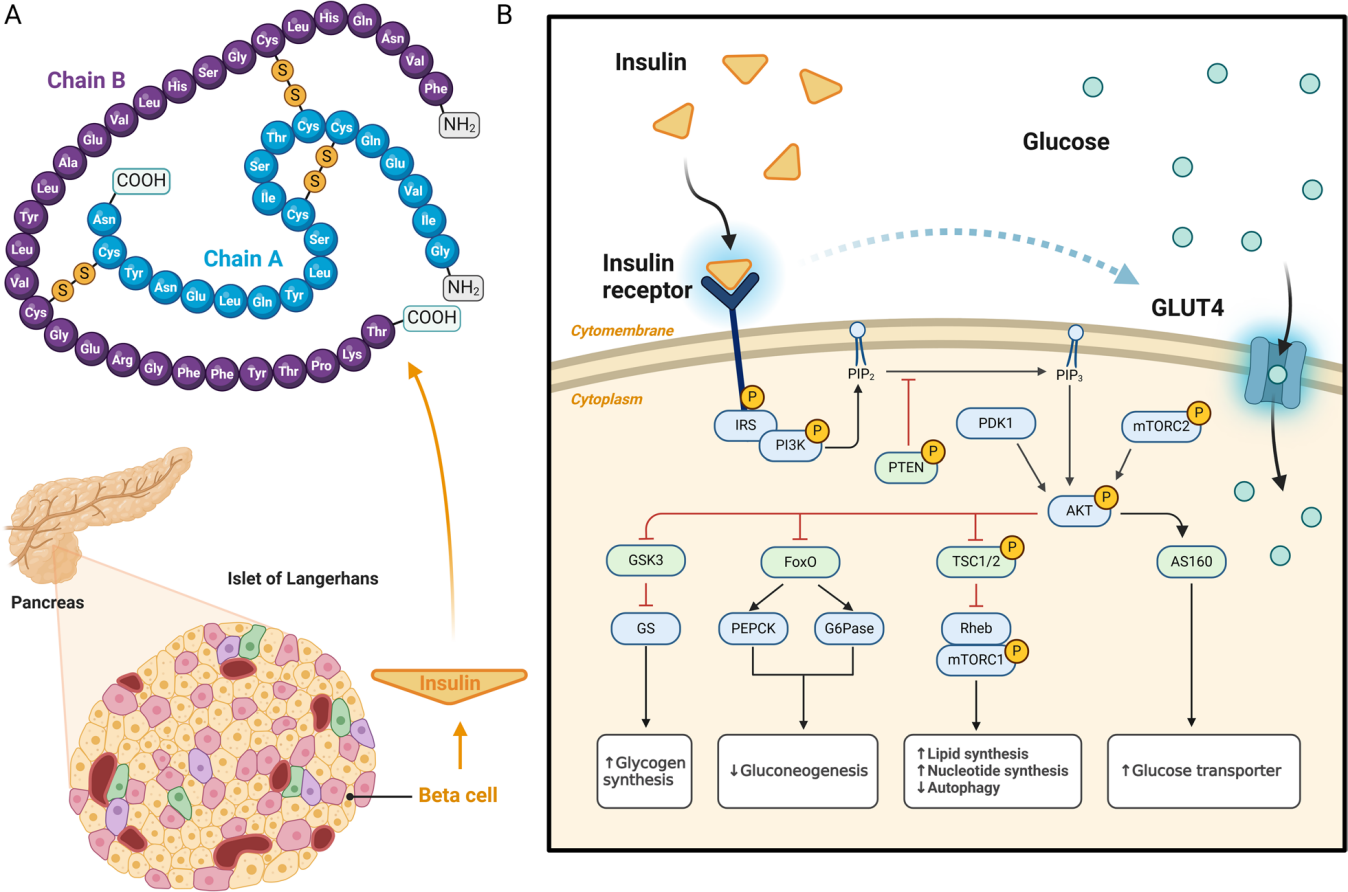
The production of insulin and the classical insulin signaling pathway. **A**, The production and structure of insulin. Insulin is synthesized by the β-cells of the islets of Langerhans in the pancreas. It consists of two polypeptide chains, linked by disulfide bonds, with a total of 51 amino acids. Insulin plays a crucial role in the regulation of glucose homeostasis, ensuring the proper balance of blood glucose levels in the body. **B **Classical insulin signaling pathway. In the classical insulin signaling pathway, insulin binds to the extracellular α-subunit of the insulin receptor (InsR), leading to the dimerization and autophosphorylation of the β-subunit, which subsequently activates its kinase activity. The phosphorylated InsR then catalyzes the phosphorylation of tyrosine residues on insulin receptor substrates (IRS), which in turn recruits and activates the PI3K complex. The catalytic subunit of PI3K, p110, phosphorylates phosphatidylinositol 4,5-bisphosphate (PIP2), converting it into phosphatidylinositol (3,4,5)-trisphosphate (PIP3). The phosphatase PTEN can counteract the actions of PI3K and Akt by dephosphorylating PIP3, thus reducing Akt phosphorylation levels in the cell. It is important to note that PI3K does not directly activate Akt. Instead, PI3K interacts with signaling proteins containing PH domains, such as Akt and phosphoinositide-dependent kinase 1 (PDK1), which facilitate Akt's translocation to the cell membrane. Once at the membrane, PDK1 catalyzes the phosphorylation of Akt at Thr 308, partially activating it. However, full activation of Akt requires phosphorylation at Ser 473. This phosphorylation is carried out by the mammalian target of rapamycin (mTOR) complex 2 (mTORC2), which fully activates Akt’s enzymatic activity. Activated Akt has numerous downstream effects, including: 1) Glycogen synthase kinase 3 beta (GSK3β) regulates the activity of glycogen synthase (GS), thereby promoting glycogen synthesis. Phosphorylation of GSK3β by Akt inhibits the constitutive activity of this key kinase, resulting in the activation of GS and the deposition of glucose as glycogen. 2) Activated Akt induces the phosphorylation of Forkhead Box O1 (FoxO1), causing its translocation from the nucleus and the loss of its transcriptional activity. Glucose-6-phosphatase G-6-pase (G6Pase) and Phosphoenolpyruvate carboxykinase (PEPCK) are two rate-limiting enzymes in the gluconeogenesis pathway. FoxO1 can bind to promoter regions of the G6Pase and PEPCK genes, increasing their transcriptional activity. This enhances hepatic glucose production, leading to elevated blood glucose levels. 3) Akt directly phosphorylates Tuberous Sclerosis Complex (TSC) 2 at multiple sites, thereby reducing the inhibitory effect of the TSC1-TSC2 complex on Ras homolog enriched in brain (Rheb) and mTORC1. This leads to the activation of mTORC1 in response to insulin signaling, regulating lipid, nucleotide, and glucose metabolism. 4) The most significant correlation with systemic blood glucose control is the phosphorylation of AS160, a 160-kDa Akt substrate. AS160 regulates the translocation of glucose transporter type 4 (GLUT4) to the cell membrane, facilitating glucose uptake into muscle, adipose tissue, and certain neurons. *FoxO1* forkhead Box O1, *G6Pase* glucose-6-phosphatase G-6-pase, *GLUT4* glucose transporter type 4, *GS* glycogen synthase, *GSK3β* glycogen synthase kinase 3 Beta, *IRS* insulin receptor substrates, *InsR* insulin receptor, *PDK1* phosphoinositide-dependent kinase 1, *PEPCK* phosphoenolpyruvate carboxykinase, *PIP2* phosphatidylinositol 4,5-bisphosphate, *PIP3* phosphatidylinositol (3,4,5)-trisphosphate, *Rheb* Ras homolog enriched in brain

Insulin plays a pivotal role as a key regulatory factor in the transition from nutrient utilization to energy storage. Insulin is a hormone produced by the β-cells of the pancreatic islets of Langerhans that regulate blood glucose levels. It consists of two polypeptide chains connected by disulfide bonds, comprising 51 amino acids. Insulin exerts its effects by binding to the insulin receptor (InsR), a transmembrane glycoprotein receptor composed of two α and two β subunits (Rorsman and Ashcroft 2018). This interaction initiates a cascade of downstream signaling pathways (Fig. 1). When insulin binds to the α subunits of InsR, it induces conformational changes that activate and auto-phosphorylate several tyrosine residues in the cytoplasmic region of the β subunits. These phosphorylated residues are recognized by the phosphotyrosine-binding (PTB) domains of adapter proteins, such as insulin receptor substrates (IRS) (Petersen and Shulman 2018). Among the six mammalian IRS proteins (IRS-1, IRS-2, IRS-3, IRS-4, IRS-5, IRS-6), IRS-1 and IRS-2 are typically considered key nodes in the insulin signaling system, closely associated with the development of insulin resistance. Specifically, under conditions of obesity, stress, and inflammation, extensive serine phosphorylation of IRS-1 occurs through the action of various kinases (Machado-Neto et al. 2018; Woo et al. 2024).

#### The brain as an insulin-sensitive organ

Brain development and the regulation of neurogenic niches are critically dependent on insulin. Insulin promotes neurogenesis by regulating the proliferation, differentiation, and survival of neural stem cells (Aberg et al. 2003). Normal insulin signaling not only plays a vital role in maintaining circuit regulation and synaptic plasticity but also in controlling neuronal and glial cell metabolism and mitochondrial function (Chen et al. 2022). It is now well-established that most tissues, including the brain, express InsR and are sensitive to insulin. Evidence indicates that InsR is expressed in both neurons and glial cells, with expression levels varying across different brain regions (Kandimalla et al. 2017; Arnold et al. 2018). InsR is particularly abundant in the hypothalamus, hippocampus, cerebral cortex, and olfactory bulb, areas critical for metabolic regulation and cognitive function (Stanley et al. 2016; Milstein and Ferris 2021). Notably, neurons and glial cells express different isoforms of the InsR α subunit. Neurons express the InsR-A isoform, whereas glial cells primarily express the InsR-B isoform (Kleinridders 2016; Cai et al. 2017). Compared to InsR-B, InsR-A exhibits a higher binding affinity for insulin, with the receptor internalization rate being ~ 1–2 times greater (Rajapaksha and Forbes 2015; Pomytkin et al. 2018). Animal studies have shown that selective disruption of neuronal InsR, particularly in the hypothalamus, increased fat mass, and peripheral insulin resistance (Wardelmann et al. 2019; Porniece Kumar et al. 2021). Conversely, restoring hypothalamic insulin action can prevent diabetes.

Under normal physiological conditions, insulin readily crosses the blood–brain barrier (BBB) via receptor-mediated transport, and this transport rate can be modulated by factors such as obesity, inflammation, and AD. Studies using endothelial cell-specific InsR knockout mice confirmed that endothelial InsR is critical for insulin trans the BBB and downstream insulin signaling in the hippocampus, hypothalamus, and frontal cortex (Konishi et al. 2017). There is also evidence suggesting that the brain can produce insulin independently (Molnár et al. 2014; Mazucanti et al. 2019; Lee et al. 2020). Proinsulin is synthesized in the pancreas and processed in the endoplasmic reticulum, where specific prohormone convertases cleave the C-peptide segment, ultimately leading to the formation of mature insulin. In response to elevated blood glucose, β-cells release insulin via exocytosis. While humans and rabbits have a single insulin-encoding gene, rodents possess two. Among these, Ins II appears to be the primary insulin gene expressed in neurons (Havrankova et al. 1978; Deltour et al. 1993; Devaskar et al. 1993). In cultured rabbit neurons and glial cells, only neurons secrete insulin into the culture medium (Devaskar et al. 1994). Limited evidence suggests that prohormone convertases are evenly distributed across various regions of the brain (Dauch et al. 1993). However, the expression of these enzymes in neurons of the paraventricular nucleus and supraoptic nucleus of the hypothalamus, which are capable of processing proinsulin, partially supports the notion of insulin production within the brain (Dong et al. 1997).

### AD and T3DM

Over the past two decades, T2DM has evolved into a complex, multifactorial, and heterogeneous disease (Cefalu et al. 2022). Approximately 90–95% of all diabetes cases worldwide are classified as T2DM(2024a). Clinically, individuals are diagnosed with T2DM when there is a relative insulin deficiency (due to pancreatic β-cell dysfunction) coupled with peripheral insulin resistance (2024a).

**Fig. 2:**
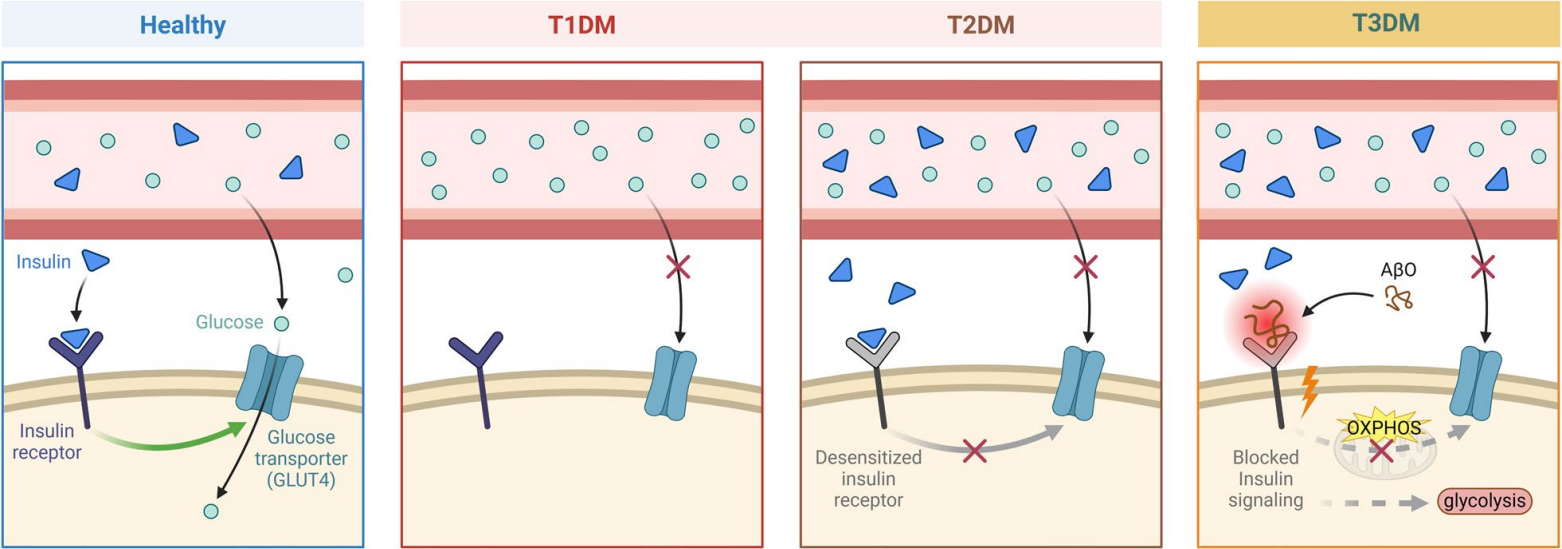
Type 1 diabetes mellitus (T1DM), Type 2 diabetes mellitus (T2DM), and Type 3 diabetes mellitus (T3DM) each have distinct characteristics. T1DM is an autoimmune disease in which the body’s immune system attacks and destroys the insulin-producing β-cells in the pancreas, leading to absolute insulin deficiency. In healthy individuals, insulin is produced in response to elevated blood glucose levels after eating, facilitating glucose uptake into cells for energy and storage. In T1DM, the lack of insulin prevents glucose absorption, resulting in hyperglycemia and inadequate cellular energy supply. The primary feature of T2DM is insulin resistance, where the body's cells do not respond properly to insulin. Over time, the pancreas may become unable to produce enough insulin to overcome this resistance. In T2DM, insulin resistance means that cells do not respond efficiently to insulin, and the pancreas struggles to meet the body’s insulin demands, leading to the accumulation of glucose in the bloodstream and the development of hyperglycemia. "T3DM" is a term used in research to describe insulin resistance in the brain, which is associated with Alzheimer’s disease (AD) but has not yet been applied clinically. In a healthy brain, insulin plays a critical role in regulating brain functions, including memory, cognition, and synaptic plasticity. However, in AD, insulin receptors (InsR) are disrupted by β-amyloid oligomers (AβO), impairing normal insulin signaling. This disruption prevents brain cells from effectively utilizing glucose, similar to the insulin resistance observed in peripheral tissues of T2DM patients. *AD* Alzheimer’s disease, *AβOs* β-amyloid oligomers, *T1DM* Type 1 diabetes mellitus, *T2DM* Type 2 diabetes mellitus, *T3DM* Type 3 diabetes mellitus, *InsR* insulin receptors

Dysregulation of insulin signaling is associated with a range of neurological disorders. More importantly, in AD, the deficits in brain insulin and insulin resistance correspond to the characteristics observed in type 1 diabetes mellitus (T1DM) and T2DM, respectively. Consequently, the coexistence of both conditions in AD has led to the conceptualization of AD as a unique form of brain-dominant diabetes, referred to as “T3DM” (de la Monte and Wands 2008; de la Monte et al. 2018) (Fig. 2).

Epidemiological data indicate that the incidence of comorbidities between T2DM and neurological diseases such as AD is high. Insulin dysregulation and alterations in glucose metabolism are considered risk factors for AD (Jiang et al. 2013; Li et al. 2015; Zhang et al. 2017). Studies have shown that T2DM increases the risk of AD by 50–100% (Grimm et al. 2016; Meng et al. 2022). In the 1990s, data from the Rotterdam Study revealed that individuals with diabetes were nearly twice as likely to develop dementia compared to age-matched non-diabetic controls (*OR* 1.9, 95% *CI* 1.2–3.1) (Ott et al. 1996). Elevated circulating blood glucose levels not only increase the risk of dementia but also accelerate the progression from mild cognitive impairment (MCI) to AD (Janson et al. 2004; Aljanabi et al. 2020). Positron emission tomography (PET) imaging using ^18^F-FDG/CT has shown significant reductions in brain glucose metabolism in MCI patients, suggesting that metabolic decline may serve as an important biomarker in the pathogenesis of AD, occurring prior to clinical manifestations of the disease (Mosconi et al. 2008).

MCI is strongly associated with systemic metabolic dysfunction and insulin resistance, including conditions such as T2DM, metabolic syndrome, polycystic ovary syndrome, and NAFLD (de la Monte et al. 2009; de la Monte 2012). Furthermore, peripheral insulin resistance in individuals without T2DM is considered a risk factor for AD within three years of diagnosis (Schrijvers et al. 2010). Notably, insulin resistance within the brain itself can occur independently of T2DM, potentially promoting or even triggering key pathological events in the disease, such as the formation of β-amyloid plaques and tau phosphorylation (Ohara et al. 2011; Kacířová et al. 2021; de la Monte 2023; Dybjer et al. 2023). This finding aligns with observed changes in the levels of insulin signaling molecules in the brains of AD patients, as well as improvements in memory following intranasal insulin administration in such cases (Claxton et al. 2015; McClure Yauch et al. 2022; Wong et al. 2024). Lastly, insulin resistance is a common feature of AD and is associated with increased amyloid plaque load, reduced hippocampal volume and cognitive function, and decreased cortical glucose metabolism, all of which correlate with diminished memory recall (Baker et al. 2011; Lee et al. 2016b; Tyagi and Pugazhenthi 2021; Kim and Arvanitakis 2023). Taken together, defects in insulin signaling and systemic insulin resistance may play significant roles in the pathogenesis of AD.

### Potential mechanisms leading to T3DM

**Fig. 3:**
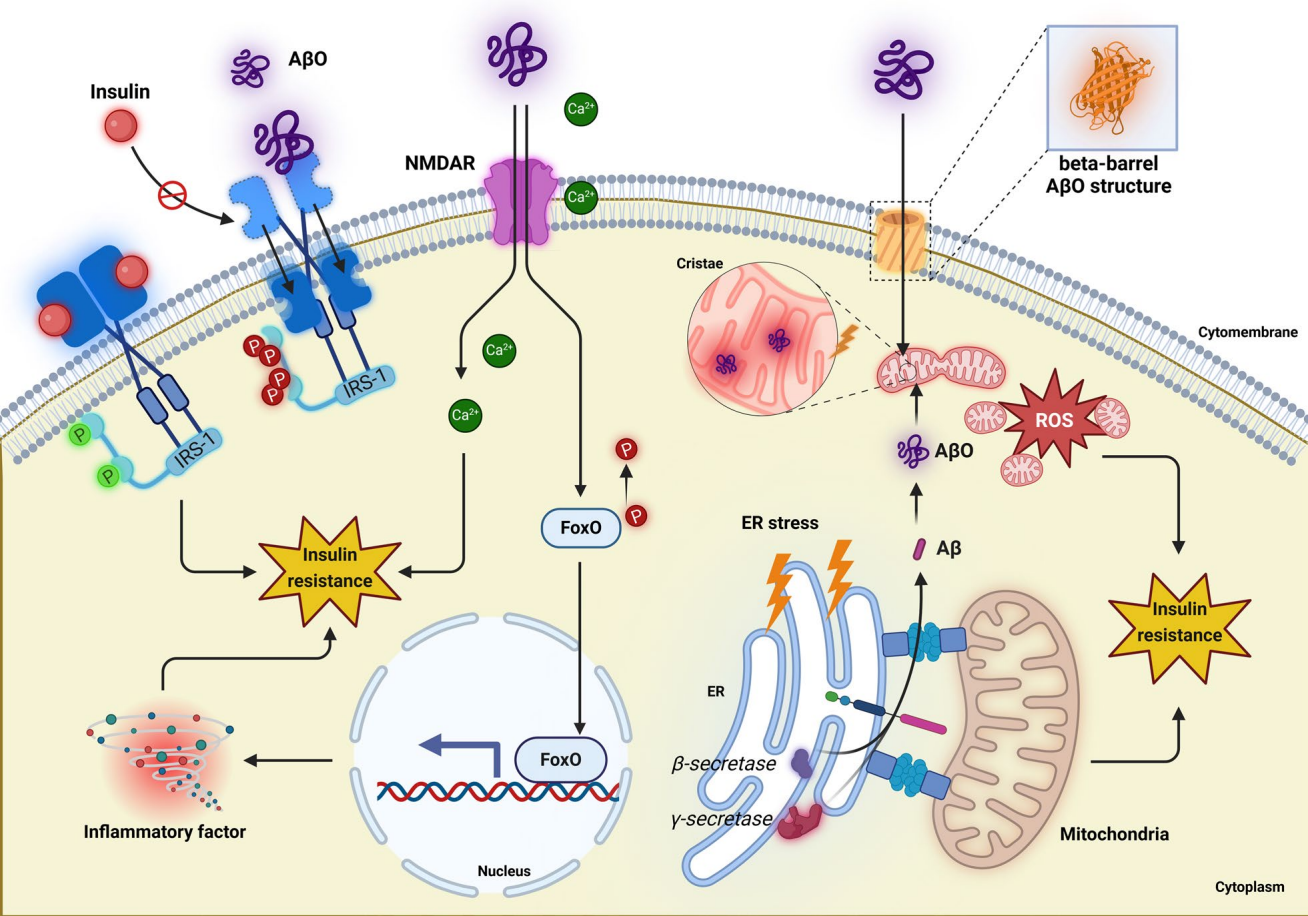
Potential mechanisms leading to T3DM. Aβ oligomers (AβOs) compete with insulin for binding to the insulin receptor (InsR), leading to the internalization of InsR and a reduction in both the affinity and the number of InsR on the cell membrane. This can also induce structural abnormalities in the target receptor that binds insulin to InsR. Furthermore, internalized InsR leads to the phosphorylation of IRS, insulin receptor substrates-1 (IRS-1) at critical serine/threonine residues, which accelerates the degradation of the phosphorylated IRS-1 protein, thereby reducing the strength of insulin signaling and promoting insulin resistance. AβO can also lead to the abnormal activation and dysregulation of N-methyl-D-aspartate (NMDA) subtype glutamate receptors (NMDARs). This aberrant activation allows for the influx of Ca^2^⁺ ions into the cell, resulting in the nuclear translocation of forkhead box O 1 (FOXO1), which ultimately triggers the generation of reactive oxygen species (ROS) and pro-inflammatory cytokines. On another front, AβO contributes to T3DM by disrupting mitochondrial function. Due to the barrel-like structure of AβO, it forms ion channels on the cell membrane that allow the oligomers to enter the cell. Aβ can also aggregate within mitochondria-associated membranes (MAMs), forming AβO aggregates. Mitochondria are one of the primary cellular targets of AβO. The presence of AβO damages the integrity of mitochondrial cristae, stimulates mitochondrial fission, and induces endoplasmic reticulum (ER) stress, thereby disrupting mitochondrial dynamics and contributing to insulin resistance. *AβOs* Aβ oligomers, *ER* endoplasmic reticulum, *FOXO1* forkhead box O 1, *InsR* insulin receptor, *IRS-1* insulin receptor substrates-1, *MAMs* mitochondria-associated membranes, *NMDA* *N*-methyl-d-aspartate, *NMDARs* *N*-methyl-d-aspartate subtype glutamate receptors, *ROS* reactive oxygen species, *T3DM* Type 3 diabetes mellitus

Aβ is a neurotoxic endogenous substance that is considered a hallmark of AD and a major trigger for T3DM. Structurally, Aβ has a hydrophilic N-terminus and a hydrophobic C-terminus (Han and He 2018). Consequently, the release of Aβ fragments initially leads to the spontaneous aggregation of Aβ monomers into soluble Aβ oligomers (AβOs), which subsequently polymerize into insoluble protofibrils that further aggregate to form amyloid plaques, also known as senile plaques (Hardy and Higgins 1992). Compared to Aβ monomers and protofibrils, the barrel-shaped structure of AβOs has a higher affinity for cell membranes, making them more likely to interact with various membrane receptors. With the InsR and the NMDAR being the most prominent (Talbot et al. 2012). In addition to binding to membrane receptors, AβOs can also induce insulin resistance by disrupting mitochondrial function (Calvo-Rodriguez and Bacskai 2021; Sayyed and Mahalakshmi 2022) (Fig. 3).

#### Impact of AβO on membrane receptors

First, AβOs compete with insulin for binding to the InsR, causing internalization of InsR and leading to abnormal phosphorylation of downstream signaling molecules, thereby disrupting insulin signaling (Schrijvers et al. 2010; de la Monte 2023). The impairment of InsR binding with insulin primarily refers to a decrease in the receptor's affinity and number on the cell membrane, or to structural abnormalities of the target receptor that hinder insulin-receptor binding (Hall et al. 2020). Generally, insulin activates InsR tyrosine kinase, which aggregates and phosphorylates various substrate-docking proteins, such as members of the IRS protein family. Among the four mammalian IRS proteins (IRS-1, IRS-2, IRS-3, IRS-4), IRS-1 and IRS-2 are considered key nodes in the insulin signaling system, and their dysfunction is closely linked to the development of insulin resistance (Tanti and Jager 2009). Mechanistically, crosstalk between downstream nucleotide-binding oligomerization domain 1 (NOD1) effectors and the insulin receptor pathway may inhibit insulin signaling by reducing IRS function (Rivers et al. 2019). Indeed, although InsR levels in T2DM models are reduced by approximately 90%, both defects in InsR and downstream signaling are implicated in the development of insulin resistance (James et al. 2021).

Evidence shows that exposure of primary hippocampal neurons to AβOs in vitro results in the loss of insulin sensitivity, inhibitory phosphorylation of IRS-1, and decreased InsR expression on dendritic membranes (Zhao et al. 2008; De Felice et al. 2009). APP/PS1 mice also exhibit impaired insulin signaling in the hippocampus, as evidenced by increased phosphorylation of IRS-1 at the serine 616 site (Bomfim et al. 2012). Phosphorylation of IRS-1 at key serine/threonine residues accelerates the degradation of the phosphorylated IRS-1 protein, thereby attenuating the intensity of insulin signaling (Draznin 2006). Abnormal phosphorylation of IRS-1 results in decreased sensitivity of insulin binding to InsR and a partial translocation of IRS-1 from the membrane to the cytoplasm, which is a major molecular basis for insulin resistance (Draznin 2006). Mechanistically, AβOs may induce or exacerbate neuronal insulin resistance through the aberrant activation of the TNF-α/JNK (tumor necrosis factor α/c-Jun N-terminal kinase) pathway, leading to serine phosphorylation of IRS-1 and inducing mitochondrial oxidative stress (Kaminsky et al. 2015; Mullins et al. 2017). Similarly, the TNF-α/JNK pathway is also activated in T2DM, contributing to peripheral insulin resistance, β-cell apoptosis, and increased oxidative stress (Li et al. 2015; Mittal and Katare 2016).

Additionally, AβOs can cause abnormal activation of NMDAR (De Felice et al. 2007; Shankar et al. 2007a; Decker et al. 2010; Paula-Lima et al. 2011a). Dysregulated NMDAR function may play a role in the impairment of neuronal insulin signaling in AD, as the AβO-induced inhibition of insulin receptor signaling can be blocked by memantine (Zhao et al. 2008). Under physiological conditions, synaptic NMDAR activity exerts antioxidant effects by inhibiting FOXO1 in the hippocampus (Papadia et al. 2008; Mubarak et al. 2009). However, in AD, dysfunctional NMDAR activity and insulin resistance may lead to nuclear translocation of FOXO1, ultimately increasing ROS generation (Manolopoulos et al. 2010). This, in turn, may further exacerbate impaired insulin signaling and neuronal dysfunction. Another possibility is that excessive NMDAR activation and Ca^2+^ influx, triggered by AβOs, stimulate the activity of tyrosine phosphatases on IRS-1, thereby weakening insulin signaling (Shankar et al. 2007b; Zempel et al. 2010; Brito-Moreira et al. 2011; Paula-Lima et al. 2011b). These possibilities are consistent with the described IR regulation mechanisms and suggest a potential physiological feedback loop between dysregulated neuronal activity and insulin signaling.

#### Mitochondrial function

AβOs can specifically translocate via the translocator of the outer mitochondrial membrane (TOM) and translocase of the inner membrane (TIM) or enter the mitochondria through mitochondrial-associated endoplasmic reticulum membranes (MAM), thereby disrupting mitochondrial function and contributing to the development of T3DM. In fact, both Aβ precursor protein (APP) and Aβ have been shown to co-localize with mitochondria and even generate within lipid raft-enriched MAM regions (Lustbader et al. 2004; Manczak et al. 2006; Wilkins 2023). Additionally, mitochondrial dysfunction contributes to insulin resistance, partly through the excessive production of reactive oxygen species ROS (Shankar et al. 2007b). In this context, insulin resistance is thought to arise from excessive mitochondrial fuel production, such as NADH and FADH_2_, without a corresponding increase in energy demand, leading to the generation and release of H_2_O_2_ from the mitochondria (Fisher-Wellman and Neufer 2012).

## Damage to mitochondrial cristae and ROS production

Mitochondrial cristae are structural folds of the inner mitochondrial membrane (IMM). The mitochondrial electron transport chain (ETC) complexes play a key role in linking cristae morphology to mitochondrial function, as these complexes are embedded within the IMM (Cogliati et al. 2013). Electron microscopy has revealed that AβOs cause severe disruption of mitochondrial cristae structures in N2a cells from mice (Manczak et al. 2010). Consistently, postmortem reports indicate that AD patients exhibit a significantly increased incidence of cristae disruption in neuronal mitochondria (Hirai et al. 2001). Cristae increases the surface area of the IMM, facilitating more efficient aerobic respiration. However, damage to cristae compromises the integrity of ETC complexes, impairing electron transfer and proton transport. This damage results in electron and proton leakage, reduced ATP production, and increased ROS generation (Lin and Beal 2006). On the one hand, ROS may induce insulin resistance by directly targeting proteins involved in glucose uptake (Anderson et al. 2009). A shift in the cellular redox state toward oxidation reduces the overall activity of serine/threonine phosphatases, which enhances the activity of stress-sensitive serine/threonine kinases and suppresses insulin signaling, thus promoting insulin resistance (Martin and McGee 2014). On the other hand, mitochondrial ROS activates inflammasomes, triggering a cascade of inflammatory responses (Zhi-Qiang et al. 2023). Many of the signaling pathways activated by inflammation involve serine/threonine kinases that can impair insulin signaling, such as JNK (Vallerie and Hotamisligil 2010). However, the precise role of mitochondrial control of inflammation in the pathogenesis of insulin resistance remains unclear, though it provides a potential mechanism through which mitochondrial dysfunction may affect insulin action.

## Mitochondrial dynamics

Mitochondrial dynamics play a crucial role in maintaining mitochondrial health, bioenergetic function, quality control, and cellular vitality. Under physiological conditions, mitochondria undergo continuous dynamic fission and fusion, resulting in morphological changes that help maintain the overall stability of the mitochondrial network. Increased Aβ production and the interaction of AβOs with dynamin-related protein 1 (Drp1) are key factors in mitochondrial fragmentation, abnormal mitochondrial dynamics, and synaptic damage (Manczak et al. 2011; Manczak and Reddy 2012). Moreover, AβOs directly induce Drp1 phosphorylation at Ser616 through Akt activation, promoting mitochondrial fragmentation and triggering downstream events, including ROS production (Kim et al. 2016).

Rats fed a high-fat diet exhibit decreased expression of mitochondrial fusion protein 2 (Mfn2) in the liver, which is accompanied by impaired insulin signaling (Lionetti et al. 2014). In contrast, overexpression of Mfn2 compensates for high-fat diet-induced disruption of insulin signaling (Gan et al. 2013). Liver-specific Mfn2 knockout mice show decreased insulin sensitivity, along with an increased degree of mitochondrial fission (Sebastián et al. 2012). Similarly, increased mitochondrial fission in skeletal muscle is associated with fat-induced insulin resistance (Luo et al. 2021). In fact, reductions in mitochondrial size and Mfn2 expression in skeletal muscle have been observed in both obesity and T2DM, and these reductions correlate with impaired insulin sensitivity (Hernández-Alvarez et al. 2010; Putti et al. 2015; Eshima 2021). All of these findings suggest that disruption of mitochondrial dynamics plays a critical role in insulin resistance and T2DM.

## Mitochondrial-endoplasmic reticulum stress coupling

MAMs are specialized subcellular compartments where the endoplasmic reticulum (ER) and mitochondria interact. These regions facilitate efficient communication between these organelles, exchanging Ca^2+^, lipids, and other metabolites to maintain cellular metabolism and integrity (Degechisa et al. 2022). MAMs exhibit lipid raft characteristics, which create a favorable environment for the γ-secretase activity of APP, emphasizing MAM as a potential site for Aβ production near the mitochondria (Li et al. 2023b). *Tubbs* and colleagues demonstrated in OB/OB mice and diet-induced insulin resistance mouse models that the integrity of MAMs is essential for insulin signaling. They showed that genetic or pharmacological inhibition of cyclophilin D (CypD) disrupted MAM integrity in both mouse and human primary hepatocytes, altering insulin signaling (Tubbs et al. 2014). Conversely, overexpression of CypD enhanced MAM integrity and improved insulin signaling in diabetic mouse liver cells. Shinijo et al. showed that palmitic acid-treated HepG2 cells exhibited a significant reduction in Ca^2+^ transfer from the ER to mitochondria and a decrease in ACSL4 (another MAM marker), suggesting that MAM disruption plays a crucial role in palmitic acid-induced insulin resistance (Shinjo et al. 2017). Furthermore, overexpression of Mfn2 partially restored MAM contact sites and improved palmitic acid-induced insulin resistance, enhancing Akt Ser473 phosphorylation (Shinjo et al. 2017). These findings underscore the important role of mitochondrial dynamics regulator Mfn2 in maintaining MAM integrity and function and highlight the potential interaction between mitochondrial fusion/fission processes and the ER in modulating insulin resistance.

## Neuronal metabolic reprogramming

Metabolic reprogramming refers to the process by which cells alter their energy production pathways to adapt to changing physiological or pathological demands (Han et al. 2021). This concept is particularly significant in cancer biology, as exemplified by the Warburg effect (Samudio et al. 2009). The Warburg effect describes a phenomenon in cancer cells where energy production predominantly relies on aerobic glycolysis, even in the presence of sufficient oxygen, instead of the more efficient OXPHOS process typically utilized by normal cells. This metabolic shift, first described by German biochemist Otto Warburg in the 1920s, is a hallmark of many cancers (Koppenol et al. 2011). However, with advances in our understanding of tumor and stem cell metabolism, metabolic reprogramming is no longer synonymous with the Warburg effect but broadly refers to any alterations in cellular metabolic mechanisms.

The Embden-Meyerhof-Parnas (EMP) pathway, encompassing glycolysis and the Krebs cycle (also known as the tricarboxylic acid (TCA) cycle), forms the core metabolic route supplying biochemical precursors for biosynthesis and energy production (Peretó 2011). In neurons, glucose is metabolized through glycolysis or the pentose phosphate pathway (PPP), followed by the TCA cycle and OXPHOS, yielding water, carbon dioxide, and ATP (Magistretti and Allaman 2015). pyruvate dehydrogenase complex (PDC) plays a pivotal role at the crossroads of glycolysis and the TCA cycle, regulating the entry of carbohydrate-derived carbon into mitochondria (Patel et al. 2014). In human eukaryotic cells, the PDC is composed of three core catalytic components: pyruvate dehydrogenase (E1), dihydrolipoamide transacetylase (E2), and dihydrolipoamide dehydrogenase (E3). In addition, it is regulated by two key enzymes: pyruvate dehydrogenase kinase (PDK) and pyruvate dehydrogenase phosphatase (PDP) (Patel et al. 2014). These enzymes modulate PDC activity through the phosphorylation (inhibition) and dephosphorylation (activation) of serine residues at positions 293, 300, and 232 on the E1α subunit of the heterotetrameric pyruvate dehydrogenase enzyme (Tovar-Méndez et al. 2005). This regulation controls the aerobic conversion of pyruvate to acetyl-CoA or its anaerobic conversion to lactate.

Neurons primarily use glucose as their energy source, oxidizing it almost completely through continuous glycolysis and the OXPHOS-associated TCA cycle (Zheng et al. 2016). However, under certain conditions, such as hypoglycemia, neurons can metabolize glutamate and glutamine to generate energy (McKenna 2007). Relatively few studies have explored the metabolic changes in neurons during insulin resistance.

### Insulin resistance and metabolic reprogramming

**Fig. 4:**
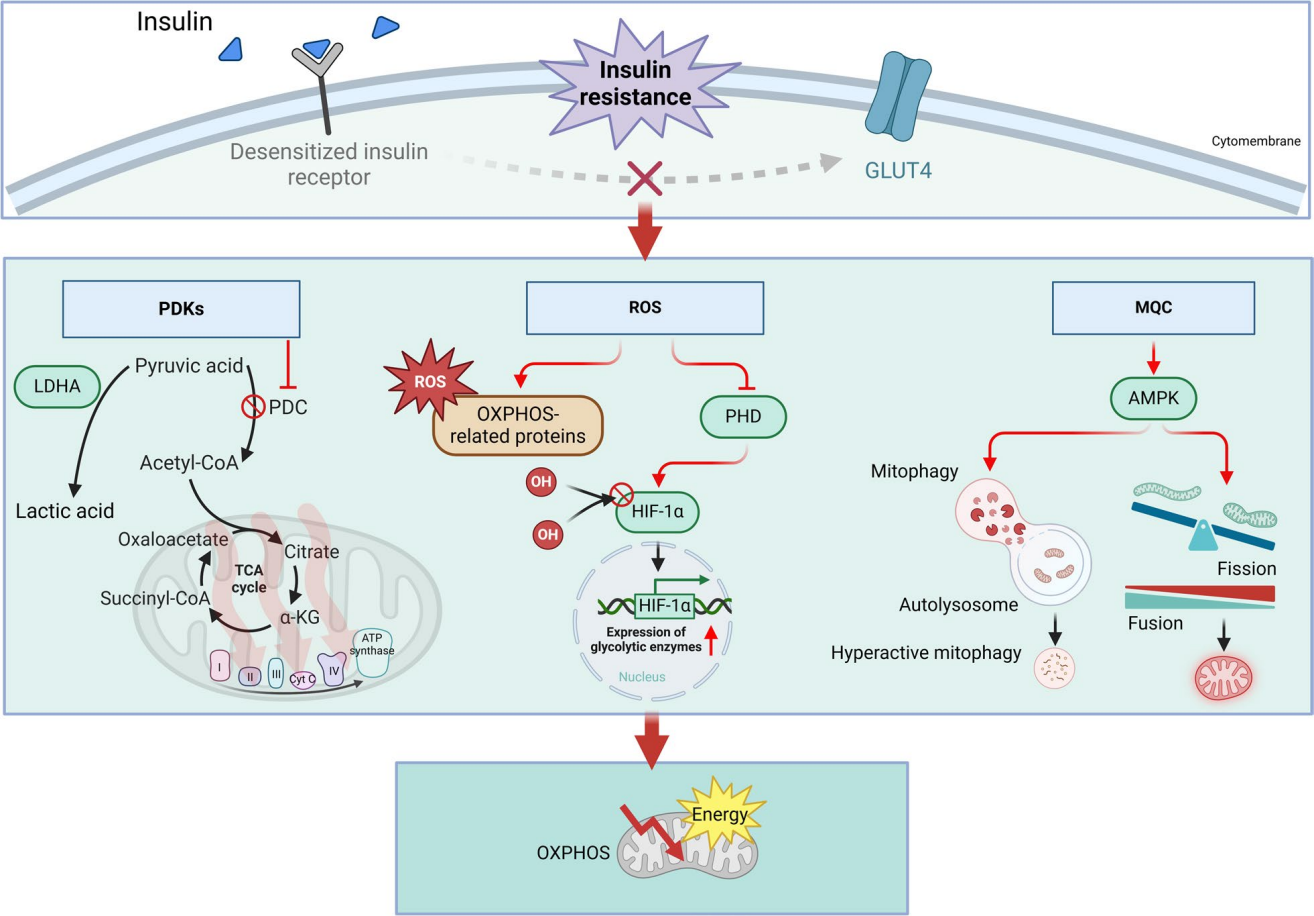
Potential mechanisms of metabolic reprogramming induced by insulin resistance. Insulin resistance activates pyruvate dehydrogenase kinases (PDKs), which inhibit the activity of pyruvate dehydrogenase complex (PDC). This suppression reduces the conversion of pyruvate to acetyl-CoA through PDC-mediated decarboxylation, thereby decreasing tricarboxylic acid (TCA) cycle flux and adenosine triphosphate (ATP) production. Furthermore, insulin resistance leads to an increase in reactive oxygen species (ROS), which not only induces oxidative modifications of key proteins involved in oxidative phosphorylation (OXPHOS) but also results in the inactivation of prolyl hydroxylases (PHDs). This causes the dephosphorylation of hypoxia-inducible factor-1α (HIF1α) and promotes the expression of enzymes associated with glycolysis. Finally, insulin resistance induces sustained activation of AMP-activated protein kinase (AMPK). On one hand, this disrupts mitochondrial dynamics, leading to mitochondrial fragmentation, which is detrimental to OXPHOS. On the other hand, prolonged mitochondrial damage activates the PINK1-Parkin pathway, continuously marking damaged mitochondria for degradation. This results in excessive mitochondrial autophagy, reducing the number of healthy mitochondria and impairing OXPHOS efficiency. *I* Complex I, *II* Complex II, *III* Complex III, *IV* Complex IV, *ATP* adenosine triphosphate, *AMPK* AMP-activated protein kinase, *Cyt C* cytochrome *C*, *HIF1α* hypoxia-inducible factor-1 alpha, *LDHA* lactate dehydrogenase A, *OXPHOS* oxidative phosphorylation, *PDKs* pyruvate dehydrogenase kinases, *PDH *pyruvate dehydrogenase, *PHDs* prolyl hydroxylases, *PINK1* PTEN-induced putative kinase 1, *ROS* reactive oxygen species, *TCA* tricarboxylic acid

Clinically, metabolic syndrome is a pathological state characterized by the clustering of various metabolic abnormalities, collectively constituting a complex syndrome of metabolic disorders. The primary cause of metabolic syndrome is insulin resistance, marked by decreased efficiency in glucose utilization. Insulin resistance also leads to compensatory shifts in energy metabolism in metabolic syndrome. This leads to compensatory shifts in energy metabolism, collectively termed metabolic reprogramming, underscoring the intricate link between insulin resistance and energy metabolism. Metabolomics data indicate that patients with T2DM exhibit elevated serum levels of pyruvate, lactate, and citrate compared to controls, suggesting enhanced glycolysis and disrupted TCA cycle function (Messana et al. 1998; Lee et al. 2018b). Similarly, Sas et al. found significantly higher urinary concentrations of glycolysis products such as lactate, phosphoenolpyruvate, 2,3-diphosphoglycerate, and glyceraldehyde-3-phosphate in T2DM patients, alongside elevated TCA cycle intermediates such as pyruvate, citrate, succinate, fumarate, and malate (Sas et al. 2016). However, these levels gradually declined as the disease progressed. Furthermore, evidence from cerebrospinal fluid (CSF) analysis reveals distinct metabolic disturbances in T2DM, including elevated levels of alanine, leucine, valine, tyrosine, lactate, and pyruvate, alongside reduced histidine levels compared to controls. These findings highlight the metabolic dysregulation associated with insulin resistance (Lin et al. 2019). Several potential mechanisms by which insulin resistance induces metabolic reprogramming are summarized below (Fig. 4).

### Potential mechanisms underlying metabolic reprogramming in insulin resistance

#### Insulin resistance and PDK activation

PDKs inhibit PDC activity, thereby regulating metabolic pathways. Among the four PDK isoforms, PDK4 exhibits the highest kinase activity, whether or not it associates with the L2 or E2p/E3BP core (Wynn et al. 2008). Aberrant regulation of PDC in diabetes involves two isoforms: PDK2 and PDK4. Both are significantly upregulated in T2DM patients and animal models fed a high-fat diet (Holness et al. 2000; Rosa et al. 2003; Spriet et al. 2004; Sikder et al. 2018). Pharmacological inhibition of PDK4, such as through dichloroacetate, has been shown to improve hyperglycemia (Crabb et al. 1981; Park et al. 2021). Diseases associated with shifts from glucose to fatty acid utilization for energy production, such as diabetes and fasting states, also upregulate PDK4 expression (Kim et al. 2023). Transcriptomics and single-cell sequencing data suggest that *Pdk4* is upregulated in AD models and the brains of AD patients, identifying it as a potential shared gene between T2DM and AD (Rasche et al. 2008; Wei 2020; Mathys et al. 2024).

Insulin plays a critical role in regulating PDK2 and PDK4 expression. It achieves this through downstream targets such as FOXO and peroxisome proliferator-activated receptor gamma coactivator 1-alpha (PGC-1α), which are primary modulators of PDK2 and PDK4 expression (Jeong et al. 2012; Shrivastav et al. 2013; Zhang et al. 2014). In mice fed a high-fat diet, insulin resistance activates PGC-1α and estrogen-related receptor alpha (ERRα), which bind to the PDK4 promoter, enhancing its mRNA and protein expression (Wende et al. 2005; Rinnankoski-Tuikka et al. 2012). Additionally, insulin inhibits PDK2 and PDK4 expression via the PI3K/Akt pathway, which phosphorylates FOXO.

Further research demonstrates that insulin reduces PDK4 gene expression by downregulating three promoter elements, including glucocorticoid response elements (GREs), FOXO1 binding sites, and ERR elements (Connaughton et al. 2010). Other regulators of PDK expression include growth hormone, adiponectin, adrenaline, and rosiglitazone, which exhibit tissue-specific effects (Attia et al. 2010; Jeong et al. 2012). PDK4 is a critical regulator of PDC activity, pyruvate oxidation, and glucose homeostasis. Knockout models of PDK4 show reduced blood glucose levels and hepatic gluconeogenesis (Jeoung et al. 2006). During fasting and diabetes, PDK4 is widely upregulated in major tissues, in response to insulin depletion and increased glucocorticoids and free fatty acids (Nakae et al. 2002). However, direct evidence linking PDK4 to AD remains limited.

#### ROS-induced metabolic reprogramming

The inefficient glucose utilization in insulin resistance is closely tied to oxidative damage. Insulin resistance contributes to oxidative stress via multiple mechanisms, including the production of advanced glycation end-products (AGEs), and ER stress and inflammation (Petersen and Shulman 2018). Excessive ROS production disrupts mitochondrial function, impairing OXPHOS activity while exacerbating insulin resistance, and creating a vicious cycle.

Insulin resistance fosters ROS generation through several pathways. Hyperglycemia induces non-enzymatic glycation of proteins and lipids, forming AGEs, which interact with AGE receptors (RAGE), stimulating oxidative stress (Li et al. 2022b). Additionally, insulin resistance often involves ER stress, characterized by impaired protein folding (Brown et al. 2020). This activates the unfolded protein response (UPR), which enhances ROS production through mechanisms such as JNK pathway activation (Bhattarai et al. 2021). Chronic low-grade inflammation further exacerbates ROS production, as inflammatory cytokines like TNF-α and Interleukin-6 (IL-6) impair insulin signaling by activating serine kinases that phosphorylate IRS and recruit immune cells such as macrophages to adipose tissue, amplifying local ROS levels (Hotamisligil et al. 1994; Cawthorn and Sethi 2008; Fazakerley et al. 2023).

ROS also act as molecular signals, promoting a shift from OXPHOS to glycolysis by stabilizing hypoxia-inducible factor-1 alpha (HIF1α) (Guzy et al. 2005; Semenza 2017). HIF1α drives anaerobic glycolysis and suppresses OXPHOS. Prolyl hydroxylase (PHD) is a key oxidative stress-sensitive inhibitor of HIF-1α. Under normoxic conditions, PHD induces the hydroxylation of proline and asparagine residues within the oxygen-dependent degradation domain of HIF-1α, leading to its degradation (Lee et al. 2016a). However, elevated levels of ROS can inactivate PHD through redox-dependent dimerization, thereby stabilizing HIF-1α even under normoxic conditions. This results in a shift from OXPHOS to anaerobic glycolysis. Consequently, the accumulation of lactate, a byproduct of anaerobic glycolysis, leads to a significant reduction in pyruvate and ATP production (Lee et al. 2016a). On one hand, insufficient supply of pyruvate—an essential substrate for the TCA cycle—disrupts its homeostasis. On the other hand, this shift results in energy depletion, compromising cell survival, particularly in high-energy-demanding cells.

Excessive ROS production also causes oxidative modifications of key enzymes involved in glycolysis and OXPHOS. Redox proteomic analyses of AD brain tissues have shown oxidative modifications of glycolytic enzymes, including aldolase, triosephosphate isomerase, glyceraldehyde-3-phosphate dehydrogenase, phosphoglycerate mutase 1, and α-enolase, in affected brain regions (Butterfield and Boyd-Kimball 2018). Additionally, oxidative modifications of aconitase (a key iron-sulfur enzyme in the TCA cycle), creatine kinase (an enzyme that helps maintain ATP levels in neurons), and ATP synthase in brain mitochondria have been observed in both MCI and AD patients (Kanski et al. 2002; Butterfield and Boyd-Kimball 2018). Furthermore, oxidative damage to mitochondrial DNA may impair energy production, and studies suggest that defects in Sirtuin 3 can exacerbate oxidative damage in AD mitochondria (Santos et al. 2012; Lee et al. 2018a). Indeed, mitochondrial dysfunction and insulin resistance are closely linked.

#### Insulin resistance and decline in mitochondrial quality

Mitochondrial quality control (MQC) refers to the processes that maintain the integrity, functionality, and quantity of mitochondria within cells (Pickles et al. 2018). On one hand, a decline in mitochondrial quality is associated with impaired pyruvate transport into mitochondria, weakening the TCA cycle. On the other hand, in the absence of sufficient mitochondrial ATP production, insulin-resistant cells often shift to glycolysis (glucose breakdown for energy) as an alternative energy source. Key components of MQC include mitochondrial biogenesis, fission, fusion, and mitophagy.

Insulin signaling is known to involve the activation of Akt, which leads to the assembly of mTORC1. mTORC1 plays a critical role in regulating protein, lipid, and fatty acid synthesis, as well as mitochondrial metabolism (Szwed et al. 2021). Through its regulation of PGC-1α and nuclear respiratory factors 1 and 2 (NRF1/2), mTORC1 is essential for mitochondrial oxidative metabolism and biogenesis. When activated by insulin, mTORC1 stimulates the production of nuclear-encoded mitochondrial proteins, integrating them into various mitochondrial metabolic pathways, including the TCA cycle, fatty acid β-oxidation, and the electron transport chain (Yoon 2017).

A second complex, mTORC2, also involves mTOR and mediates Akt activation, which negatively regulates FoxO1 (Jais et al. 2014; Jung et al. 2019). FoxO1 promotes transcription of heme oxygenase-1 (HO-1), triggering a metabolic shift from OXPHOS to glycolysis in neurons. When MQC declines, cells experience a pseudo-hypoxic state, characterized by low oxygen utilization despite sufficient oxygen availability, which activates HIF1α (Li et al. 2023c). FoxO1 forms a regulatory loop with HIF-1α, upregulating glycolysis-related enzymes such as hexokinase (HK), phosphofructokinase, and pyruvate kinase, thereby enhancing glucose metabolism via glycolysis (Li et al. 2023c). Furthermore, HIF-1α promotes the expression of lactate dehydrogenase (LDH), which converts pyruvate into lactate, enabling glycolysis to proceed even when mitochondrial oxidative capacity is diminished (Kshitiz et al. 2022).

The decline in MQC reduces the number of functional mitochondria available for OXPHOS, diminishing the cell's ability to oxidize glucose for ATP production. As mitochondrial ATP output decreases, the cell's adenosine monophosphate (AMP) /ATP ratio increases, activating AMPK (Herzig and Shaw 2018). AMPK promotes glycolysis, inhibits anabolic processes such as protein synthesis, and enhances catabolic pathways, including autophagy and mitophagy (Herzig and Shaw 2018). These processes can further reduce mitochondrial content while temporarily compensating for energy deficits by increasing glucose uptake and glycolytic flux. However, this also reinforces the metabolic shift toward glycolysis. Additionally, AMPK promotes mitochondrial fission, disrupting mitochondrial dynamics (Chen et al. 2019). Studies have shown increased mitochondrial fission in the hippocampus in response to insulin resistance (Ruegsegger et al. 2019). High glucose levels and impaired insulin signaling create an energy imbalance, leading to a mismatch between cellular energy production and demand (Chan 2020). This results in an energy deficit and dysregulated mitochondrial dynamics, characterized by reduced expression of fusion proteins and increased fission proteins. While this adaptation increases mitochondrial numbers, it also produces fragmented, dysfunctional mitochondria. Inhibiting excessive mitochondrial fission with Drp1 inhibitors helps restore mitochondrial dynamics and prevents insulin resistance in high-fat diet-induced mouse models (Filippi et al. 2017). For example, reducing Drp1 activity in primary hippocampal neurons isolated from OB/OB mice improved ATP production associated with obesity-induced deficits (Huang et al. 2015). Treatment with the Drp1 inhibitor mdivi-1 restored hippocampal synaptic plasticity, linking excessive mitochondrial fission to cognitive deficits associated with insulin resistance (Huang et al. 2015).

In the context of insulin resistance, the clearance of damaged mitochondria in insulin-sensitive cells relies on mitophagy. However, persistent mitochondrial damage can lead to sustained activation of the PINK1 (PTEN-induced putative kinase 1) -Parkin pathway, continuously tagging damaged mitochondria for degradation (Li et al. 2022a). This overactivation of mitophagy can decrease mitochondrial quality by failing to effectively remove dysfunctional mitochondria, impairing OXPHOS. Both excessive and impaired mitophagy negatively impact neuronal survival (Tang et al. 2024). Studies show that silencing *Parkin* or inhibiting PINK1 translation can restore mitochondrial OXPHOS (Liu et al. 2021; Wang et al. 2021a; Huang et al. 2023b). Although most research indicates that neuronal autophagy is inhibited in AD, some studies suggest that low-dose Aβ_1-40_ triggers mitochondrial quality decline and mitophagy activation, reducing ATP production (Li et al. 2022a). Notably, mitophagy alterations in metabolic diseases may follow a time-dependent pattern, with initial activation followed by later inhibition (Hombrebueno et al. 2019). Considering that Aβ accumulates in autophagosomes of dystrophic neurites and serves as a major intracellular reservoir of toxic peptides in the AD brain, future studies should cautiously interpret these findings (Jin et al. 2017).

## Treatment strategies

Currently, treatments for both T2DM and AD are primarily symptomatic and target-specific, presenting significant challenges in disease prevention and control. Moreover, the severe side effects of existing pharmacological treatments have driven scientists to seek alternative therapeutic strategies. It is well established that insulin resistance is a hallmark of T3DM, with overlapping but distinct pathological features connecting diabetes, insulin resistance, and cognitive decline. Studies using AD mouse models demonstrate that improving insulin signaling in the brain can ameliorate disease symptoms. Consequently, various approaches have been explored to address insulin signaling defects in AD, including exogenous insulin supplementation, enhancing insulin sensitivity, improving mitochondrial function to boost OXPHOS, and employing supplementary or alternative interventions. A table has been compiled based on clinical data registered on *ClinicalTrials.gov* to facilitate an appropriate analysis of treatment strategies for T3DM. Further details can be found in the identifiers listed in the table (Table 1).

**Table 1:** Clinical trials under recruitment or ongoing were summarized according to different targets

Study title	Target	Intervention/treatment	Duration	Recruitment status	Phase	Identifier
Effect of Physiologic Insulin Administration on Cognition	Exogenous insulin infusion	Pulsed insulin clamps (40 mU/m^2^/min insulin infusion)	2 Weeks	Enrolling by invitation	Not Applicable	NCT06424652
Combination of Intranasal Insulin With Oral Semaglutide to Improve Cognition and Cerebral Blood Flow: a Feasibility Study	Exogenous insulin infusion	Treatment: Group 1 Will receive intranasal insulin therapy as well as Oral Semaglutide Sham Comparator: Group 2 Will receive active intranasal insulin therapy and placebo Oral Semaglutide Sham Comparator: Group 3 Will receive intranasal insulin placebo and active Oral Semaglutide Placebo Comparator: Group 4 Will receive intranasal insulin placebo and Oral Semaglutide placebo	6 and 12 Months	Recruiting	II	NCT06072963
Device Study for Intranasal Delivery of Insulin	Exogenous insulin infusion	Insulin first, then placebo Participants will be randomly assigned to receive regular insulin (U100, 20 IU) administered with an intranasal nebulizer-like device. Participants in this arm will then receive placebo at visit 3 during second intervention period	30 min	Active, not recruiting	II	NCT03857321
Dapagliflozin Effect in Cognitive Impairment in Stroke Trial	Insulin enhancers	Treatment: Dapagliflozin 10 mg PO q24h for 12 months plus standard treatment with statins, platelet antiaggregant, and hypoglycemic medications Active Comparator: Standard treatment Standard treatment with statins, platelet antiaggregant, and hypoglycemic medications	6 and 12 Months	Recruiting	II, III	NCT05565976
Evaluating Liraglutide in Alzheimer's Disease	Insulin enhancers	Treatment: Liraglutide Daily administration of 1.8 mg liraglutide by subcutaneous injection Placebo Comparator: Placebo Daily administration of matched placebo by subcutaneous injection	12 Months	Unknown status	II	NCT01843075
Metformin in Alzheimer's Dementia Prevention	Insulin enhancers	Treatment: metformin Extended-release metformin 500 mg tablets up to 2,000 mg (4 tablets) a day once at night. The maximum dose will be attempted during a titration period in the first month of the study Placebo: Placebo Placebo tablets identical to extended-release metformin 500 mg tablets up to 4 tablets a day once at night. The maximum dose will be attempted during a titration period in the first month of the study	18 Months	Active, not recruiting	II, III	NCT04098666
A Randomised Double-blind Placebo-controlled Clinical Trial Investigating the Effect and Safety of Oral Semaglutide in Subjects With Early Alzheimer´s Disease	Insulin enhancers	Treatment: Oral semaglutide 14 mg Participants are given oral semaglutide once daily Placebo: Placebo Participants are given oral placebo once daily	0–104 Weeks	Active, not recruiting	III	NCT04777409
Evaluating the Effects of Liraglutide, Empagliflozin and Linagliptin on Mild Cognitive Impairment Remission in Patients With Type 2 Diabetes: a Multi-center, Randomized, Parallel Controlled Clinical Trial With an Extension Phase	Insulin enhancers	Treatment: Liraglutide Liraglutide will be titrated from 0.6 mg/day to a final dose 1.8 mg/day during the first 2 weeks, if well tolerated Treatment: Empagliflozin Empagliflozin will be initiated and maintained at 10 mg/ day every morning until the completion of the study Treatment: linagliptin linagliptin will be initiated at 5 mg/ day every morning	0–48 Weeks	Recruiting	Not applicable	NCT05313529
Sirtuin-NAD Activator in Alzheimer's Disease	Mitochondria	Treatment: MIB-626 Subjects will either take MIB-626 or placebo tablet twice a day for 90 days. For those who receive MIB-626, give subjects 1000 mg of the drug, twice a day for 90 days. MIB-626 will be in two 500 mg tablets Placebo Comparator: Placebo Tablet Subjects will be randomized to receive either the placebo or MIB-626 tablets twice a day orally	90 Days	Recruiting	I, II	NCT05040321
Causal Effect of Coenzyme Q10 Nutrition and Cognitive Dysfunction in the Metabolic Storm (Hyperglycemia and Sarcopenia) and Brain-derived Neurotrophic Factor	Mitochondria	Treatment: Coenzyme Q10 Coenzyme Q10 300 mg/day (150 mg/b.i.d.) Placebo Comparator: Dextrin	12 Weeks	Recruiting	Not applicable	NCT06040905
Effects of Nicotinamide Riboside on Bioenergetics and Oxidative Stress in Mild Cognitive Impairment/ Alzheimer's Dementia	Mitochondria	Treatment: Mild Cognitive Impairment and Alzheimer's Dementia Participants will take 4 pills every day, each containing 250 mg NR (NIAGEN^®^ by Chromadex; www.chromadex.com), via the oral route, for 12 weeks	0, 6, 12 Weeks	Recruiting	I	NCT04430517
The Mito-Frail Trial: Effects of MitoQ on Vasodilation, Mobility and Cognitive Performance in Frail Older Adults	Mitochondria	Treatment: MitoQ capsule Capsules containing MitoQ (5 mg/capsule) totaling 20 mg taken every day for 12 weeks Placebo: Placebo capsule Gelatin capsules	0, 12 Weeks	Recruiting	II	NCT06027554
Exploring Nasal Drop Therapy With Small Extracellular Vesicles for ALS	Stem cells and regenerative medicine	Treatment: Exosomes Patients in this arm will receive exosomes derived from human umbilical cord blood mesenchymal stem cells as a nasal drop, administered once daily, twice a week, for a total of two weeks Placebo: Exosomes placebo Patients in this arm will receive a placebo nasal drop mimicking exosomes derived from human umbilical cord blood mesenchymal stem cells, administered once daily, twice a week, for a total of two weeks	24 h, 4 ± 1 Weeks	Recruiting	I, II	NCT06598202
Neurologic Stem Cell Treatment Study	Stem cells and regenerative medicine	Autologous bone marrow aspiration and separation of Bone Marrow Derived Stem Cell fraction then provided intravenously and intranasally (lower 1/3 of nasal passages)	0,1,3,6 and 12 Months	Recruiting	Not applicable	NCT02795052
The Impact of 6-months of Resistance Training on Brain and Muscle Health in Older Adults With MCI	Exercise	Experimental: Intervention Progressive resistance training of lower limb muscles. Frequency of intervention: 2–3 times per week. Duration of intervention: 24 weeks Experimental: Active control Flexibility training of the lower limb muscles. Frequency of intervention: 2–3 times per week. Duration of intervention: 24 weeks	0, 12, 24, 48, 72 Weeks	Recruiting	Not applicable	NCT06252844
Physical Activity, Alzheimer's Disease and Cognition Relative to APOE Genotype	Exercise	Experimental: Physical Activity Condition Subjects will be asked to attend virtual exercise sessions 3 times a week for 1 year No Intervention: Usual Care Control Participants in the usual care control will maintain their normal health practices for 1 year	0, 6, and 12 Months	Active, not recruiting	Not applicable	NCT03876314
Time Restricted Eating in Alzheimer's Disease	Diet intervention	Experimental: Interventional Participants will be instructed to follow a 16/8 regimen characterized by 16 h of fasting and an 8-h eating window daily, on approximately 5 days/week, for 3 months	0 and 3 Months	Recruiting	Not applicable	NCT06429124
The Ketogenic Diet for Alzheimer's Disease	Diet intervention	Experimental: Modified Atkins 2:1 Ketogenic diet The Atkins 2: 1 diet as prescribed for the participants of the intervention group is based on a diet moderately rich in protein (meat, fish, cheese, eggs, vegetable proteins) and without restriction of fats, provided they are balanced, but limiting the carbohydrate intake (bread, pasta, rice) to 50 g / day. The ratio calories from fat / calories from protein + carbohydrates will be 3 to 1 No Intervention: Control diet	12 Months	Recruiting	Not applicable	NCT04701957
Therapeutic Diets in Alzheimer's Disease	Diet intervention	Experimental: Ketogenic Diet Study partners will be instructed to assist participants in adherence to a 1:1 ketogenic diet (approximately 70% fat, < 10% carbohydrate, and 20% protein as energy) Active Comparator: Therapeutic Lifestyles Changes Diet The diet consists of 20–35% fat, 50–60% carbohydrate, and ~ 15% protein as energy. Fat intake will comprise < 7% saturated fat, ≤ 20% monounsaturated fat, and ≤ 10% polyunsaturated fat as total energy. Cholesterol consumption will be ≤ 200 mg per day. Participants are encouraged to eat ≥ 2 servings of fruit and ≥ 5 servings of vegetables per day	0 and 12 Weeks	Recruiting	Not applicable	NCT03860792

### Targeting insulin resistance

#### Insulin infusion

Although there is a steep gradient between plasma and CSF insulin levels in healthy individuals, the transport of insulin from plasma to CSF is slow (Strubbe et al. 1988; Schwartz et al. 1990; Mazucanti et al. 2019). Even after pharmacological elevation of plasma insulin levels for four hours, CSF insulin concentrations remain below the typical fasting plasma insulin levels (Wallum et al. 1987). This suggests that reversing brain insulin resistance through peripheral insulin supplementation is challenging. While systemic high-dose insulin therapy is a viable clinical option for treating T2DM patients, it is unsuitable for addressing brain insulin resistance in AD patients or individuals without diabetes due to the risk of hypoglycemia (McCall et al. 2023).

Interestingly, limited studies have reported that intravenous insulin infusion, while maintaining plasma glucose at fasting baseline levels, can significantly improve memory in AD patients (Craft et al. 1996). However, increasing peripheral insulin levels has the potential to deplete brain insulin-degrading enzyme, a key enzyme responsible for degrading Aβ in the brain (Qiu and Folstein 2006; Tian et al. 2023). This depletion could negatively impact Aβ degradation, counteracting the intended therapeutic effects. Therefore, supplementing peripheral insulin to enhance brain insulin signaling is not an optimal solution, and increasing brain insulin levels remains a significant challenge. To address this issue, researchers have explored intranasal insulin delivery, which bypasses the blood–brain barrier and directly transports insulin to the brain via the cavernous sinus capillaries into cerebral circulation. Preliminary studies indicate that intranasal insulin delivery holds promise for improving cognitive function and AD biomarkers (Reger et al. 2008; Craft et al. 2012, 2017, 2020). These findings suggest that intranasal insulin could offer a more effective and targeted approach to addressing brain insulin resistance in AD.

#### Insulin sensitizers

The degree of brain insulin resistance varies significantly among individuals and is challenging to quantify. Merely increasing brain insulin levels may enhance the binding of insulin to its receptor, potentially causing downregulation of available InsR and exacerbating insulin resistance (Bar et al. 1976). As an alternative to intranasal insulin administration, insulin receptor sensitizers can enhance insulin-InsR binding or its downstream effects through various mechanisms (Storozhevykh et al. 2007; Storozheva et al. 2008; Miller et al. 2011). Currently, metformin and thiazolidinediones (TZDs) are considered promising candidates for treating AD and other forms of dementia. A meta-analysis revealed that these insulin sensitizers reduce the combined relative risk of dementia in diabetic patients by 22% during combined therapy. When used as monotherapies, metformin and TZDs independently reduce dementia risk by 21% and 25%, respectively (Basutkar et al. 2023). Additionally, liraglutide, a glucagon-like peptide-1 (GLP-1) receptor agonist, has been shown to restore hippocampal insulin responsiveness via the InsR/IRS-1/Akt pathway and improve working memory in APP/PS1 transgenic mice (Talbot and Wang 2014). Although treatment with this drug for six months improved brain glucose uptake in patients, no differences were observed in amyloid plaque deposition or cognitive function compared to the placebo. Currently, liraglutide remains in the preclinical research phase (Gejl et al. 2016).

### Targeting metabolic reprogramming

#### Targeting PDKs

Mounting evidence suggests that energy metabolism alterations associated with neurological disorders play a central role in their molecular neuropathophysiology (An et al. 2018; Mullins et al. 2018; Weise et al. 2018; Kellar and Craft 2020). The functional link between cytosolic glycolysis and mitochondrial OXPHOS places PDC at the core of mitochondrial metabolism. Impaired PDC activity, whether due to natural aging or acquired diseases, exhibits similar pathological patterns, emphasizing PDC and its regulatory kinases as critical therapeutic targets for a range of neurological conditions.

Dichloroacetate (DCA), a pyruvate mimetic compound and specific PDK inhibitor, promotes a metabolic shift from glycolysis to OXPHOS, facilitating pyruvate oxidation within mitochondria (Wang et al. 2021b; Schoenmann et al. 2023). DCA administration has been shown to reduce elevated lactate levels in both serum and CSF (Stacpoole et al. 2003). Furthermore, DCA has been reported to alleviate symptoms such as abdominal pain, headaches, and stroke-like episodes while also improving cognitive function in patients (Saitoh et al. 1998).

In addition to DCA, several other PDK inhibitors have demonstrated the potential to enhance PDC activity and restore ATP levels across various organs. These include SDZ048-619, AZD7545 (a selective PDK2 inhibitor), diisopropylamine dichloroacetate (a PDK4 inhibitor), and phenylbutyrate (Morrell et al. 2003; Ferriero et al. 2013; Yamane et al. 2014). These compounds have been shown to significantly improve PDC activity. Similarly, FX11, a small-molecule inhibitor of LDHA, can pharmacologically inhibit PDK via reducing lactate production, thereby mitigating inflammation and chronic pain driven by diabetic neuropathy (Rahman et al. 2016; Han et al. 2023). These additional PDK inhibitors, along with approaches that reduce lactate accumulation or neutralize its effects, represent promising pharmacological tools. They provide a foundation for further exploration of glucose oxidation stimulation as a therapeutic target for various neurological diseases.

#### Targeting mitochondria

Mitochondrial OXPHOS enzymes play a pivotal role in metabolic reprogramming by regulating ATP production, ROS signaling, redox balance, and the availability of biosynthetic precursors. By modulating OXPHOS enzyme activity, cells can reconfigure their metabolic pathways to meet physiological demands, adapt to stress, or shift between catabolic and anabolic states. Coenzyme Q_10_ (CoQ_10_), a critical component of the ETC, functions as an electron acceptor to promote ATP generation and acts as an antioxidant within the mitochondrial matrix and inner membrane (Wang et al. 2024). Several studies have identified CoQ_10_ as a potential therapeutic target for AD, stabilizing mitochondria damaged by neurotoxins and oxidative stress, and improving memory and behavioral performance in Tg19959 and APP/PS1 mouse models of AD (Dumont et al. 2011; Muthukumaran et al. 2018). Targeted mitochondrial antioxidants, such as MitoQ, a modified CoQ_10_ molecule, offer enhanced therapeutic potential. Unlike CoQ_10_, MitoQ is a smaller molecule with improved cellular uptake and an affinity for negatively charged mitochondria. MitoQ treatment has been shown to prevent cognitive decline and oxidative stress in 3 × Tg AD mice, extend lifespan, improve ETC function, and protect mitochondrial cardiolipin content (McManus et al. 2011; Young and Franklin 2019).

Given that ROS are byproducts of OXPHOS, mitigating ROS accumulation while maintaining ATP production is a critical focus. Alpha-lipoic acid (LA), an essential cofactor for PDC and alpha-ketoglutarate dehydrogenase (α-KGDH), has been shown to enhance mitochondrial function, activate antioxidant responses, reduce ROS generation, and improve insulin sensitivity (Dieter et al. 2022). LA can cross the BBB, reduce Aβ-induced neuronal damage, and induce Akt expression, underscoring its neuroprotective effects mediated partially through PKB/Akt signaling (Della Giustina et al. 2017). Similarly, β-hydroxybutyrate targets mitochondrial complex I to reduce ROS levels, induce ATP production in brain mitochondria and improve memory in AD patients (Tieu et al. 2003; Maalouf et al. 2007).

Polyphenols, a diverse class of plant-derived secondary metabolites with antioxidant properties, are abundant in fruits, vegetables, tea, and red wine. Dietary supplementation with polyphenols or monomeric phenols has been extensively studied for AD prevention and treatment. For example, resveratrol, a polyphenol found in red wine and grapes, and epigallocatechin gallate (EGCG), found in green tea, activate AMPK and Sirtuin 1, which upregulate PGC-1α, the master regulator of mitochondrial biogenesis (Teixeira et al. 2019). This activation promotes new mitochondrial formation and improves cellular energy metabolism. The combination of resveratrol and EGCG has been shown to regulate mitochondrial biogenesis and restore mitochondrial OXPHOS (He et al. 2022; Meng et al. 2023). Furthermore, polyphenols like gallic acid, an LDH inhibitor, have been demonstrated to suppress lactate production by inhibiting pyruvate-to-lactate conversion, thereby enhancing OXPHOS efficiency (Han et al. 2015).

### Stem cells and regenerative medicine

Stem cell and regenerative medicine strategies hold promise for metabolic reprogramming in AD through mechanisms such as improving insulin signaling, reducing inflammation, and exerting autocrine/paracrine effects. Brain-derived neurotrophic factor (BDNF), a neurotrophic factor secreted by stem cells, binds to tropomyosin receptor kinase B (TrkB), activating the IRS1/2, PI3K, and Akt pathways (Bathina and Das 2015; Żebrowska et al. 2018). BDNF has been shown to lower blood glucose levels to normal in db/db mice in a dose-dependent manner and increase pancreatic insulin levels (Nonomura et al. 2001). However, the delivery of neurotrophic factors is not the primary benefit of stem cell therapy for neurodegenerative diseases. While neurotrophic factor delivery appears less effective in reversing neurodegeneration, it can enhance the therapeutic efficacy of stem cell transplantation (Duncan and Valenzuela 2017).

A healthy neurovascular system is essential for delivering insulin to brain cells. Stem cells can promote neurovascular repair, facilitating insulin and glucose transport to support metabolic reprogramming in AD (Vargas-Rodríguez et al. 2023). Stem cell therapies have also shown promise in restoring BBB integrity, thereby improving the delivery of insulin and other nutrients to the brain. Additionally, stem cells can transfer healthy mitochondria to the brain (Liu et al. 2023). Studies indicate that mitochondrial transfer not only restores bioenergetics but also reprograms the metabolic state of recipient cells, enabling them to adapt to stress or environmental changes (Brestoff et al. 2021; Korpershoek et al. 2022; Patel et al. 2023). This approach highlights the potential for developing therapeutic strategies targeting mitochondrial dysfunction in disease contexts.

### Other therapeutic approaches

Exercise is one of the most potent regulators of peripheral insulin resistance and has emerged as an active area of research for preventing AD and cognitive decline. Physical activity has been shown to reduce the risk of AD (Stephen et al. 2017; Song 2023). Specifically, exercise mitigates cognitive deficits by improving cerebral blood flow and metabolism (Stojanovic et al. 2020). In rodent studies, exercise enhanced brain insulin sensitivity, improved mitochondrial function, reduced oxidative stress, and decreased tau hyperphosphorylation and aggregation in neurons (Jeong et al. 2018; Ruegsegger et al. 2019). Furthermore, a three-month aerobic exercise program was found to promote neurogenesis and cognition in AD patients by increasing brain ketone transport, effectively improving cerebral energy metabolism (Castellano et al. 2017).

Dietary interventions are effective modulators of peripheral insulin resistance and AD. Among the most notable is the DASH diet (Dietary Approaches to Stop Hypertension) (Agarwal et al. 2023). Adhering to the DASH diet has been shown to improve fasting insulin levels, which are associated with better cognitive performance (van den Brink et al. 2019). Additionally, a randomized controlled trial (RCT) demonstrated that cognitively normal adults and individuals with MCI who followed either a high-fat, high-simple carbohydrate diet or a low-saturated fat, low-sugar isocaloric diet for four weeks experienced distinct outcomes (Bayer-Carter et al. 2011). The high-fat, high-sugar diet decreased cerebrospinal fluid insulin concentrations, shifting healthy adults toward patterns commonly observed in AD patients, whereas the low-fat, low-sugar diet increased insulin concentrations in MCI patients to levels comparable to healthy controls.

Moreover, caloric restriction has shown significant benefits for a wide range of chronic conditions, including obesity, T2DM, cardiovascular diseases, cancer, and neurodegenerative brain disorders. A meta-analysis of 12 RCTs involving 545 participants revealed that intermittent fasting significantly reduced body mass index (BMI) and fasting glucose levels (Cho et al. 2019). Animal studies further indicate that intermittent fasting improves memory by enhancing hippocampal insulin signaling and inhibiting Aβ deposition in AD models (Shin et al. 2018). The potential mechanism underlying the benefits of intermittent fasting involves periodic metabolic shifts, wherein the body transitions from hepatic glucose metabolism to fatty acid-derived ketone body production. During fasting, intermediates of the TCA cycle are redirected toward gluconeogenesis—a liver-dominant process for glucose synthesis. As a result, acetyl-CoA accumulates and is diverted into the ketogenesis pathway, producing ketone bodies that are exported from the liver. In the brain, ketone bodies are metabolized to generate acetyl-CoA, which inhibits the PDC, preserving pyruvate. This preserved pyruvate, a key glycolytic intermediate, further suppresses glycolysis, reducing the rate of glucose metabolism. Ketone bodies also modulate the expression and activity of various proteins and molecules crucial for health and aging, including PGC-1α, fibroblast growth factors, NAD^+^, sirtuins, poly(ADP-ribose) polymerase 1 (PARP1), and ADP-ribosyl cyclase. These factors are closely associated with the pathophysiology of neurodegenerative diseases, highlighting the therapeutic potential of metabolic reprogramming (Puchalska and Crawford 2021).

## Conclusions and prospects

As research into metabolic diseases deepens, metabolic reprogramming is no longer confined to oncology. Convincing evidence suggests that abnormal glucose metabolism is a critical component of AD pathology and progression. However, the mechanisms underlying glucose metabolism defects and their detrimental downstream effects on cellular function and survival remain at an early stage of investigation. This review analyzed the molecular links between insulin resistance and AD, explored how insulin resistance drives metabolic reprogramming in neurons, and summarized therapeutic strategies targeting insulin resistance and energy metabolism.

Despite these advances, several key questions remain. First, how does AβO interact with InsR, and how does this affect downstream insulin signaling pathways? Current understanding is limited to clinical observations. Given the small spatial constraints of AβO's tubular binding interface, which offers flexibility for small-molecule interactions, future research should integrate chemical, computational, and biological approaches to address this question. Second, how can brain insulin levels be accurately quantified? Clinical methods currently rely on functional magnetic resonance imaging (fMRI) to measure whole-brain cerebral blood flow (CBF) as a proxy for brain insulin sensitivity (Kullmann et al. 2022; Li et al. 2023a). However, these methods have low temporal resolution and are costly. Identifying specific biomarkers to streamline clinical assessment and improve sensitivity is imperative. Finally, any metabolic reprogramming events in the brain, whether pathological or therapeutic, must be approached with caution. Both neurons and glial cells are metabolically sensitive and may exert beneficial or detrimental effects on AD progression.

In summary, understanding the connections between insulin resistance, metabolic reprogramming, and AD remains a complex challenge. However, these efforts hold significant promise for advancing early diagnosis, effective prevention, and therapeutic interventions for AD.

## Data Availability

No datasets were generated or analysed during the current study.

## Abbreviations

α-KGDH: Alpha-ketoglutarate dehydrogenase
Aβ: β-Amyloid
AβOs: Aβ oligomers
AD: Alzheimer's disease
AGEs: Advanced glycation end-products
AMP: Adenosine monophosphate
AMPK: AMP-activated protein kinase
APP: Aβ precursor protein
ATP: Adenosine triphosphate
Bad: Bcl-2-associated death promoter
BBB: Blood–brain barrier
BDNF: Brain-derived neurotrophic factor
BMI: Body mass index
CBF: Cerebral blood flow
CNS: Central nervous system
CoQ_10_: Coenzyme Q_10_
CypD: Cyclophilin D
DASH: Dietary approaches to stop hypertension
DCA: Dichloroacetate
Drp1: Dynamin-related protein 1
EGCG: Epigallocatechin gallate
EMP: Embden-Meyerhof-Parnas
ER: Endoplasmic reticulum
ETC: Electron transport chain
FDA: Food and Drug Administration
fMRI: Functional magnetic resonance imaging
FOXO: Forkhead box O
GLP-1: Glucagon-like peptide-1
GREs: Glucocorticoid response elements
GSK3β: Glycogen synthase kinase-3β
HIF1α: Hypoxia-inducible factor-1 alpha
HK: Hexokinase
HO-1: Heme oxygenase-1
IL-6: Interleukin-6
IMM: Inner mitochondrial membrane
InsR: Insulin receptor
IRS: Insulin receptor substrates
JNK: C-Jun N-terminal kinase
LA: Alpha-lipoic acid
LDH: Lactate dehydrogenase
MAM: Mitochondrial-associated endoplasmic reticulum membranes
MCI: Mild cognitive impairment
Mfn2: Mitochondrial fusion protein 2
MQC: Mitochondrial quality control
mTORC2: Mammalian target of rapamycin complex 2
NAFLD: Non-alcoholic fatty liver disease
NMDA: *N*-Methyl-d-aspartate
NMDAR: *N*-Methyl-d-aspartate receptor
NOD1: Nucleotide-binding oligomerization domain 1
NRF1/2: Nuclear respiratory factors 1 and 2
OXPHOS: Oxidative phosphorylation
PARP1: Poly(ADP-ribose) polymerase 1
PDC: Pyruvate dehydrogenase complex
PDK: Pyruvate dehydrogenase kinase
PDK1: 3-Phosphoinositide-dependent protein kinase 1
PDP: Pyruvate dehydrogenase phosphatase
PET: Positron emission tomography
PGC-1α: Peroxisome proliferator-activated receptor gamma coactivator 1-α
PH: Pleckstrin homology
PHD: Prolyl hydroxylase
PI3K: Phosphoinositide 3-kinase
PINK1: PTEN-induced putative kinase 1
PIP2: Phosphatidylinositol-4,5-bisphosphate
PIP3: Phosphatidylinositol-3,4,5-trisphosphate
PPP: Pentose phosphate pathway
PTB: Phosphotyrosine-binding
RAGE: AGE receptors
RCT: Randomized controlled trial
ROS: Reactive oxygen species
SH2: Src homology 2
T1DM: Type 1 diabetes mellitus
T2DM: Type 2 diabetes mellitus
T3DM: Type 3 diabetes mellitus
TCA: Tricarboxylic acid
TIM: Translocase of the inner membrane
TNF-α: Tumor necrosis factor alpha
TOM: Translocator of the outer mitochondrial membrane
TrkB: Tropomyosin receptor kinase B
TSC2: Tuberous sclerosis complex 2
TZDs: Thiazolidinediones
UPR: Unfolded protein response
